# Dermoscopic Nail Disorders in School-Going Children

**DOI:** 10.7759/cureus.36848

**Published:** 2023-03-29

**Authors:** Fatima Zahoor, Arfan ul Bari, Najia Ahmed, Tariq M Malik, Syed Arbab Shah, Ghazal Afzal

**Affiliations:** 1 Department of Dermatology, PNS (Pakistan Navy Station) Shifa Hospital, Karachi, PAK; 2 Department of Dermatology, Combined Military Hospital/Army Medical College, Rawalpindi, PAK

**Keywords:** anemia, nutritional deficiency, onychoscopy, nail eczema, childhood nail disorder, dermoscopic

## Abstract

Introduction

The majority of nail diseases in children are comparable to those in adults, while there are some physiological changes that start to happen around this age and go away over a few years. These conditions could be symptoms of infections and systemic illnesses. Pediatric nail disorders are typically easy to diagnose clinically, although there are occasionally conditions that masquerade as juvenile nail problems. Dermoscopy has grown in favour as a rapid, easy, non-invasive clinical procedure for examining nail diseases. This study aims to assess dermoscopic findings of child nail diseases.

Methods

A prospective analysis was conducted for seven months between January and July, 2022, at PNS Shifa Hospital, Karachi, Pakistan. A total of 180 patients who presented in the outpatient department with any dermatological complaint underwent thorough history and examination. Special emphasis was given to clinical and dermoscopic examinations of nails. Data analysis enclosed descriptive and inferential statistics. The quantitative data was presented with help of mean and standard deviation, while the qualitative data was presented with help of frequency and percentage tables. Chi-square test was applied to compare nail findings diagnosed on clinical examination and dermoscopic examination.

Results

The mean age of study subjects was 9.4±3.2 years and ranged between five years and 18 years. The most common nail changes were hand eczema (n=41, 23%) followed by nail changes due to nutritional disorders (n=38, 21%), anaemia (n=34, 19%) and habit tic deformity (n=31, 17.2%).

Conclusions

Clinical evaluation is crucial for nail disease diagnosis. Dermoscopy of nails can help with the final diagnosis of nail disease and confirm clinical diagnoses. Also, it aids in the management of nail illnesses by providing a clearer picture of pathology and nail structure.

## Introduction

The ability to scratch, handle small items, and protect fingers from physical harm are all benefits of having healthy nails, in addition to their importance for aesthetic reasons [1]. Nail diseases may result in sociopsychological issues that lower the quality of life. Although most nail abnormalities in children are identical to those in adults, some physiological changes do take place during this period and pass with time. Disorders of the nails may indicate a systemic illness, such as infections, dermatoses and congenital/inherited diseases [2].

Although the clinical diagnosis of paediatric nail issues is frequently simple, there are also disorders that manifest as paediatric nail issues. Skin biopsy in young children may require specific procedures, such as sedation/general anaesthesia, even though histological investigation can validate the clinical diagnosis. Several skin imaging methods have been developed recently to bridge the gap between clinical and histological assessment of skin lesions [3]. Dermoscopy has already become a well-liked clinical tool for examining nail diseases, for the reason that it is rapid, easy, and non-invasive. Dermoscopy has been demonstrated to be quite useful for the non-invasive diagnosis of a number of dermatological conditions [4].

The nail matrix, nail plate, nail bed, hyponychium, proximal nail fold, and distal margin of the nail plate can all be evaluated via nail dermoscopy. It is a crucial tool for completing clinical examinations when evaluating a number of nail diseases, including nail pigmentations and infectious, inflammatory, and traumatic nail disorders. A video dermoscope, which provides magnifications of up to 200 times, or a portable dermoscope can be used for dermoscopic examination [5,6].

Although there are several studies investigating the nail findings with the naked eye or histopathologically in children with nail disorders, studies with dermoscopy are limited [5,7]. Besides, visualizing nail findings using a dermatoscope that improves the clinicians’ diagnostic accuracy significantly enhances results in comparison to examination with the naked eye. Dermoscopic examination has gained preference and is now a routine method for examining pediatric nail findings [8]. Clinically the most common nail findings are pitting, hyperkeratosis, splinter haemorrhages, oil drops, longitudinal grooves, leukonychia, and onychoschizia [9]. On the other hand, the most common dermoscopic features of nails have been reported in a limited number of studies, most of which have been performed in the adult population [9-17]. In this research, we aimed to evaluate and describe dermoscopic findings of nail disorders in children.

The majority of studies done locally or internationally on dermoscopic nail findings in children could not prove the significance of this method over clinical examination alone. The paucity of information in this regard exists in our part of the world (eastern and southern Asia), which has a different geographical setup, climate, dietary habits, lifestyles, and economical soundness as compared to the western world. With this information in mind, we planned this study. Due to the scarcity of data, the work done on this subject would also add benefits in framing policies for such patients in the future.

## Materials and methods

This is a prospective cross-sectional descriptive study performed over seven months (January-July 2022). The study was approved by the Ethical Committee PNS Shifa, Karachi, Pakistan (approval number: ERC/2021/DERMA/05). A total of 180 patients who presented in the outpatient department of PNS Shifa Hospital, Karachi, Pakistan, with any dermatological complaints were enrolled in the study using a non-probability sampling technique after taking written informed consent from parent/guardian.

Openepi calculator (Open Source Epidemiologic Statistics for Public Health, version 3.01 updated 2006) was used to calculate the sample size by taking the prevalence of nail disorders in children, which is about 11% [13], a margin of error is equal to 5%, the confidence interval was 95%; the calculated sample size was 180. Those with clinically noticeable nail unit involvement and aged between three to 18 years of either gender irrespective of any duration of disease were included. Those receiving therapy for the primary reason already and those who refused to give consent were eliminated.

Two senior consultants made the diagnosis, primarily based on clinical characteristics. Basic demographic data, including age, sex, duration of disease, and co-morbidities were noted. To identify any underlying illnesses, all patients had clinical examinations. In order to find any dermatological conditions linked to nail abnormalities, patients underwent examinations. To diagnose any nail diseases, all patients underwent an inspection of 20 of their nails in accordance with the established protocol. The nail diseases were photographed using a smartphone's camera. A handheld polarised Delta 20 dermoscope (HEINE Optotechnik GmbH & Co. KG, Gilching, Germany) was used to evaluate each nail's dermoscopic condition, and an iPhone 12 (Apple Inc., Cupertino, California, United States) was used to capture digital dermoscopic photographs of nails. The connection fluid between the contact plate and the nail plate was ultrasound gel.

The data were acquired and entered using Microsoft Excel (Microsoft Corporation, Redmond, Washington, United States). IBM SPSS Statistics for Windows, Version 26.0 (Released 2019; IBM Corp., Armonk, New York, United States) served as the analysis programme. Both descriptive and inferential statistics were used in data analysis. Mean and standard deviation were used to portray quantitative data, and frequency and percentage tables were used to illustrate qualitative data. The nail results determined by clinical examination and dermoscopic examination were compared using chi-square test.

## Results

The mean age of study participants was 9.4±3.2 years and ranged between five years and 18 years with the mean duration of the disease being 2±1.2. Demographic characteristics and frequency of different nails areshown in Table 1.

**Table 1:** Demographic and nail details of the patients (n=180)

Demographic Details	Frequency n (%)
Age (mean + SD)	9.4±3.2
Duration of disease (mean + SD)	2±1.2
Gender	
Male	96 (53.3%)
Female	84 (46.7%)
Side of nail Involvement	
Right hand finger nails	141 (78.6%)
Left hand figure nails	123 (68.4%)
Right foot toenails	78 (43.6%)
Left foot toenails	73 (40.8%)

In 180 patients with nail diseases as per clinical diagnosis, nutritional nail changes were found in 38 (21%), nail changes due to anemia in 34 (19%), nail changes in hand eczema in 41 (23%), traumatic nail changes in 22 (12.2%), habit tic deformity in 31 (17.2%), leukonychia striata (Mees lines) in 16 (9%), melanocytic navus in nine (5%), onychomycosis in 26 (14%), nail psoriasis in five (2.7%), nail lichen planus in six (3.3%) and one (0.5%) patient in each of following: glomus tumor, median nail dystrophy, nail changes in Darier disease, nail changes in ectodermal dysplasia, onychogryphosis, pachyonychia congenita, and subungual hematoma.

Of the 180 patients with nail diseases according to dermoscopic diagnosis; Nutritional nail changes were found in 38 (21%), nail changes in anemia 34 (19%), nail changes in hand eczema 41 (23%), traumatic nail changes in 22 (12.2%), habit tic deformity in 31 (17.2%), leukonychia striata (Mees lines) in 16 (9%), melanocytic navus in nine (5%), onychomycosis in 29 (16.2%), nail psoriasis in six (3.3%), nail lichen planus in six (3.3%), and one (0.5%) patient in each of following: glomus tumor, median nail dystrophy, nail changes in Darier disease, nail changes in ectodermal dysplasia, onychogryphosis, pachyonychia congenita, and subungual hematoma. There were no statistically significant differences between clinical and dermoscopic examination in the diagnosis of different nail diseases (P-value >0.05) (Table 2). Nail findings on clinical and dermoscopic examination are shown in Table 3.

**Table 2:** Comparison between clinical diagnosis and dermoscopic diagnosis of different nail diseases (n=180)

Nail Diseases	Clinical n (%)	Dermoscopic n (%)	P-value
Nutritional nail changes	38 (21%)	38 (21%)	1
Nail changes in anemia	34 (19%)	34 (19%)	1
Nail changes in hand eczema	41 (23%)	41 (23%)	1
Traumatic nail changes	22 (12.2%)	22 (12.2%)	1
Habit tic deformity	31 (17.2%)	31 (17.2%)	1
leukonychia striae (Mees lines)	16 (9%)	16 (9%)	1
Melanocytic nevus	9 (5%)	9 (5%)	1
Onychomycosis	26 (14%)	29 (16.2%)	0.5
Nail Psoriasis	5 (2.7%)	6 (3.3%)	0.5
Nail lichen planus	6 (3.3%)	6 (3.3%)	1
Glomus tumor	1 (0.5%)	1 (0.5%)	1
In-growing nail (onychocryptosis)	1 (0.5%)	1 (0.5%)	1
Median nail dystrophy	1 (0.5%)	1 (0.5%)	1
Nail changes in Darier disease	1 (0.5%)	1 (0.5%)	1
Nail changes in ectodermal dysplasia	1 (0.5%)	1 (0.5%)	1
Onychogryphosis	1 (0.5%)	1 (0.5%)	1
Pachyonychia congenita	1 (0.5%)	1 (0.5%)	1
Subungual hematoma	1 (0.5%)	1 (0.5%)	1

**Table 3:** Clinical and dermoscopic signs of different nail diseases (n=180)

Nail Findings	Clinical Examination	Dermoscopic Examination
Nutritional nail disease, 38 (21%)		
Onycholysis	13 (34%)	13 (34%)
Discoloration	13 (34%)	13 (34%)
Transverse ridges	13 (34%)	13 (34%)
Cracking	13 (34%)	13 (34%)
Nail changes in anemia, 34 (19%)		
Onycholysis	23 (67.6%)	23 (67.6%)
Leukonychia	23 (67.6%)	23 (67.6%)
Transverse ridges	23 (67.6%)	23 (67.6%)
Nail changes in hand eczema, 41 (23%)		
Onycholysis	41 (100)	41 (100)
Subungual hyperkeratosis	41 (100)	41 (100)
Splinter hemorrhage	41 (100)	41 (100)
Transverse ridges	41 (100)	41 (100)
Discoloration	41 (100)	41 (100)
Cracking	41 (100)	41 (100)
Traumatic nail disease, 22 (12.2%)		
Pitting	0 (0.00)	22 (100)
Discoloration	22 (100)	22 (100)
Dystrophy	22 (100)	22 (100)
Habit tic deformity, 31 (17.2%)		
Subungual hyperkeratosis	31 (100)	31 (100)
Longitudinal ridges	31 (100)	31 (100)
Discoloration	0 (0.0)	31 (100)
Dystrophy	31 (100)	31 (100)
Leukonychia striata (Mees lines), 16 (9%)		
Leukonychia	16 (100)	16 (100)
Discoloration	16 (100)	16 (100)
Cracking	16 (100)	16 (100)
Melanocytic navus, 9 (5%)		
Subungual hyperkeratosis	0 (0.0)	6 (66.6)
Discoloration	9 (100%)	9 (100%)
Onychomycosis, 26 (14%)		
Onycholysis	23 (89.3%)	24 (92%)
Subungual hyperkeratosis	14 (53.6%)	14 (53.6)
Splinter hemorrhage	1 (3.8%)	2 (7.6%)
Pitting	1 (3.8%)	1 (3.8%)
Paronychia	10 (39.3%)	10 (39.3%)
Transverse ridges	4 (15.3%)	4 (14.2%)
Longitudinal ridges	1 (3.8%)	1 (3.8%)
Discoloration	22 (84.6%)	22 (84.6%)
Thinning	1 (3.8%)	1 (3.8%)
Thickening	18 (69.6%)	19 (71.4)
Cracking	24 (92%)	24 (92%)
Dystrophy	2 (7.6%)	2 (7.6%)
Nail psoriasis, 5 (2.7%)		
Onycholysis	2 (40%)	2 (40%)
Subungual hyperkeratosis	3 (60%)	3 (60%)
Splinter hemorrhage	3 (60%)	3 (60%)
Pitting	4 (80)	4 (80)
Paronychia	0 (0%)	0 (0%)
Transverse ridges	2 (40%)	2 (40%)
Longitudinal ridges	1 (20%)	1 (20)
Discoloration	3 (60%)	3 (60%)
Thickening	2 (40%)	2 (40%)
Cracking	6 (100%)	6 (100%)
Dystrophy	1 (20%)	2 (40%)
Nail linchen planus, 6 (3.3%)		
Onycholysis	2 (33.3%)	2 (33.3%)
Subungual hyperkeratosis	2 (33.3%)	2 (33.3%)
Pterygium	3 (50%)	3 (50%)
Leukonychia	2 (33.3%)	2 (33.3%)
Transverse ridges	2 (33.3%)	2 (33.3%)
Longitudinal ridges	4 (66.6%)	4 (66.6%)
Discoloration	6 (100%)	6 (100%)
Thickening	3 (50%)	3 (50%)
Dystrophy	2 (33.3%)	2 (33.3%)
Cracking	4 (66.6%)	4 (66.6%)
Thinning	0 (0)	0 (0)
Glomus tumor, 1 (0.5%)		
Pitting	1 (100)	1 (100)
Leukonychia	0 (0.0)	1 (100)
Discoloration	1 (100)	1 (100)
Cracking	1 (100)	1 (100)
Ingrowing Nail (onychocryptosis), 1 (0.5%)		
Onycholysis	1 (100)	1 (100)
Subungual hyperkeratosis	1 (100)	1 (100)
Discoloration	1 (100)	1 (100)
Nail changes in Darier disease, 1 (0.5%)		
Subungual hyperkeratosis	0 (0)	1 (100)
Splinter hemorrhage	1 (100)	1 (100)
Discoloration	1 (100)	1 (100)
Thickening	1 (100)	1 (100)
V-shaped notch of nail plate	1 (100)	1 (100)
Nail changes in ectodermal dysplasia, 1 (0.5%)		
Thinning	1 (100)	1 (100)
Anonychia	1 (100)	1 (100)
Cracking	1 (100)	1 (100)
Median nail dystrophy, 1 (0.5%)		
Transverse ridges	1 (100)	1 (100)
Longitudinal ridges	1 (100)	1 (100)
Discoloration	1 (100)	1 (100)
Dystrophy	1 (100)	1 (100)
Cracking	1 (100)	1 (100)
Pachynoychia congenita, 1 (0.5%)		
Subungual hyperkeratosis	1 (100)	1 (100)
Splinter hemorrhage	0 (0.0)	1 (100)
Pitting	1 (100)	1 (100)
Discoloration	1 (100)	1 (100)
Thickening	1 (100)	1 (100)
Cracking	1 (100)	1 (100)

## Discussion

Although nail diseases are not very common in children, their involvement has importance in diagnosing multiple systemic conditions in this age group. According to estimates, 0.05-3% of infants and kids have nail problems [12]. Its prevalence varies across communities and previous investigations [13-16]. According to Iglesias et al., 11% of people under the age of 17 have nail problems [13].

The patients in the current study ranged in age from five to 18 years, with a mean value of 9.4±3.2 years. This is comparable to a study done previously by Oğrum et al., in which patients' mean ages ranged from 1-17 years, with a mean of 9.18±5.12 years [17]. The fact that males dominated our survey could be attributed to their substantially rising beauty concerns and their easier access to healthcare facilities. Almost identical observations were made by Neerja et al., in their study [18].

In our investigation, hand eczema-related nail alterations were the most prevalent. It was noted in 41 (23%) patients who were diagnosed clinically, and the same abnormalities were discovered in 55 (52.8%) patients during the dermoscopic examination. The findings corroborate those of Rathod et al., who discovered that 13% of individuals with nail disorders also had hand eczema [19]. Only 1.92% of patients were found to have hand eczema, according to El-Hamd et al. [20]. Because different populations were researched in these studies, results may vary. In addition, the studies mentioned above included both children and adults in their sample populations.

Onycholysis, subungual hyperkeratosis, splinter hemorrhage, transverse ridges, discoloration, and cracking were additional symptoms of hand eczema in 41 patients (100%). El-Hamd et al. reported the same results; however, their investigation indicated that 50% of patients had an incidence of nail abnormalities [20]. According to Kaur et al.'s findings, 17% of patients with onychomycosis experienced pain, and 100% of them had discolored nails [21], whereas Gupta et al. observed that 92% of patients with toe-nail onychomycosis experienced discoloration [22]. The modest disparity in outcomes is presumably due to some variations in study designs, anomalies taken into account, and populations assessed in investigations.

In this study, nail alterations brought on by dietary problems were the second most frequent nail condition. Based on clinical evaluation and dermoscopic examination, it was discovered in 38 (21%) patients. According to El-Hamd et al. [20] and Rathod et al. [19], they observed documented nutritional nail alterations in three (2.8%) and one (0.5%) patients, respectively. This finding is rather high. Ours is a third-world nation that is still developing economically, with a higher population of undernourished children. This variation is likely caused by several ethnic, environmental, social, and cultural factors. Due to the fact that both of these investigations were retrospective analyses, it's possible that tiny nail changes that have minimal clinical importance weren't taken into account or reported [23,24]. Onycholysis, discoloration, transverse ridges, and cracking were the most frequent nail abnormalities associated with nutritional alterations in this study (34%). These results were nearly identical to those of the study by El-Hamd et al., who discovered the same results in 33.3% of the patients [20].

In the current investigation, nail alterations brought on by anemia were the third most frequent nail problem. It was seen in 34 (9%) patients who had a clinical and dermoscopic evaluation. according to Akbaş et al., 3.7% of patients with nail problems had nail alterations indicative of anemia [9].

Onycholysis, leukonychia, and transverse ridges (n=23, 67.6% each) were the most frequent nail alterations associated with anemia. El-Hamd et al. discovered the same results in 50% of cases [20].

According to clinical and dermoscopic exams, there were further nail abnormalities in the current investigation, including traumatic nail changes (22) (12.2%), habit tic deformity (31%), leukonychia striata (Mees lines) (16%), melanocytic nevus (5%), and nail lichen planus (3.3%). Onychomycosis, however, was discovered in 29 (16.2%) of the cases and 26 (14%) of the cases upon clinical evaluation. Similarly, six (3.3%) cases of dermoscopic examination and five (2.7%) cases of clinical examination both revealed nail psoriasis.

According to clinical and dermoscopic examinations, the remaining relatively uncommon findings included one (0.5%) patient each with a glomus tumor, median nail dystrophy, nail changes due to Darier disease, nail changes due to ectodermal dysplasia, onychogryphosis, pachyonychia congenita, and subungual hematoma.

In our investigation, there were no statistically significant differences in the diagnosis of various nail conditions between clinical and dermoscopic tests. This is the first study that we are aware of that looks at the dermoscopic nail features in our community. Although there was a study done with solely pediatric patients, it is unclear whether dermoscopy was used on all patients [10]. This study's main goal is to assist dermatologists and pediatricians in identifying abnormal nails without the need for an intrusive treatment like a nail biopsy.

This study had a lot of limitations. To obtain more precise information on the diagnosis of various nail conditions, multicenter research with bigger sample numbers should be conducted. To confirm the diagnosis of nail anomalies, future multicenter studies with a bigger sample size and more diagnostic methods are advised.

## Conclusions

According to this study, nutritional nail changes, anemia-related nail changes, and habit tic deformity were the most prevalent nail illnesses, followed by nail changes in hand eczema. Although nail dermoscopy can help in the management of nail illnesses by enabling improved imaging of nail structure and pathology, clinical examination is still a key component in the diagnosis of many nail diseases.

This study further emphasises the value of nail dermoscopy as a diagnostic aid for nail abnormalities and as a means of avoiding additional nail biopsies in the paediatric population. To better explain the novel dermoscopic manifestations of nail anomalies and to confirm our early findings, future research should use bigger sample sizes and control groups.
